# Cecal dermoid masquerading dermoid cyst of ovary: a case report and review of the literature

**DOI:** 10.1186/s13256-020-02570-y

**Published:** 2021-01-30

**Authors:** Tushar Subhadarshan Mishra, Saubhagya Kumar Jena, Supriya Kumari, Suvendu Purkait, Pavithra Ayyanar, Pallabi Nayak

**Affiliations:** 1 Department of General Surgery, All India Institute of Medical Sciences, Room No 403, Academic Building, AIIMS Road, Patrapada, Sijua, Bhubaneswar, Odisha 751019 India; 2 Department of Obstetrics and Gynaecology, All India Institute of Medical Sciences, Bhubaneswar, Odisha India; 3 Department of Pathology, All India Institute of Medical Sciences, Bhubaneswar, Odisha India

**Keywords:** Case report, Cecal neoplasm, Cecum, Dermoid cyst, Mature cystic teratoma

## Abstract

**Background:**

The ovary is the most common site of occurrence of mature cystic teratomas (dermoid cysts). These are the most common ovarian germ cell tumor in the reproductive age group, accounting for 10–20% of all ovarian neoplasms, with a 1–2% risk of malignancy. A cecal dermoid cyst is a rare entity with only ten cases having been reported so far, eight of which could be retrieved as the rest were reported in different languages. None of these cases were managed laparoscopically. Here we present the first case of cecal dermoid managed laparoscopically.

**Case presentation:**

A 35-year-old nulliparous Indian Hindu woman presented with complaints of on and off abdominal pain for 10 months. The abdominal examination revealed a well-defined mass of about 10 × 5 cm size, palpable in the right iliac fossa. On sonography, it was suggestive of a right-sided ovarian dermoid cyst. The lesion measured 10 × 7 × 5 cm on a contrast-enhanced computed tomogram (CT) scan. It was well defined and hypodense and located in the right lower abdomen. The ovarian tumor markers were normal. On laparoscopy, the uterus, bilateral tubes, and ovaries were found to be healthy. The cyst was seen arising from the right medial wall of the cecum at the ileocecal junction, which was excised laparoscopically. Histopathological study revealed it to be a mature cystic teratoma.

**Conclusion:**

Ovarian mature cystic teratoma commonly has an indolent course and can present with palpable abdominal mass, pain, or vomiting due to complications like torsion, hemorrhage, or infection. Alternatively, these cysts can be asymptomatic and incidentally detected. Clinicians should be aware of the variety of presentations of dermoid cysts of the bowel as well as mesentery. The exact location of the teratoma eluded us till the laparoscopy despite adequate imaging including a contrast-enhanced CT scan having been performed preoperatively. We are reporting this as it is a rare entity, and this knowledge will help gynecologists and surgeons make an appropriate surgical decision.

## Background

Mature cystic teratomas (dermoid cysts) are the most common ovarian germ cell tumor in women of reproductive age, constituting 10–20% of all ovarian neoplasms [1, 2]. They have a 1–2% risk of malignancy [1]. Predominantly they have a midline location, like the mediastinum, nasal sinuses, and pineal gland. Rarely they may occur at locations away from the midline, like the omentum, abdominal wall, retroperitoneum, and cecum. Various theories have been postulated regarding the development of dermoid off the midline. This case of a cecal dermoid closely mimicked an ovarian dermoid, and the actual location of the lesion was missed till surgery, despite contrast-enhanced computed tomography (CECT). This case is being reported because of its rarity and its potential to elude accurate diagnosis despite adequate investigations.

## Case presentation

A 35-year-old nulliparous Indian Hindu woman presented to the obstetrics and gynecology out-patient department of our institute with intermittent complaints of pain in the right lower abdomen for 10 months. She attained menarche at 13 years of age, and her previous menstrual cycles were regular. She was married for 1 year and was trying to conceive. She had no known medical comorbidities.

On examination, she was of average built. A mass of 10 × 5 cm was palpable in the right iliac fossa, which was non-tender, firm, and mobile. The lower pole was palpable. Per speculum examination was unremarkable. Per vaginal examination revealed the uterus to be anteverted, normal-sized, firm, mobile with the left fornix free, and a mass around 10 × 5 cm size felt in the right fornix. The mass was non-tender, firm, and was separate from the uterus.

**Fig. 1:**
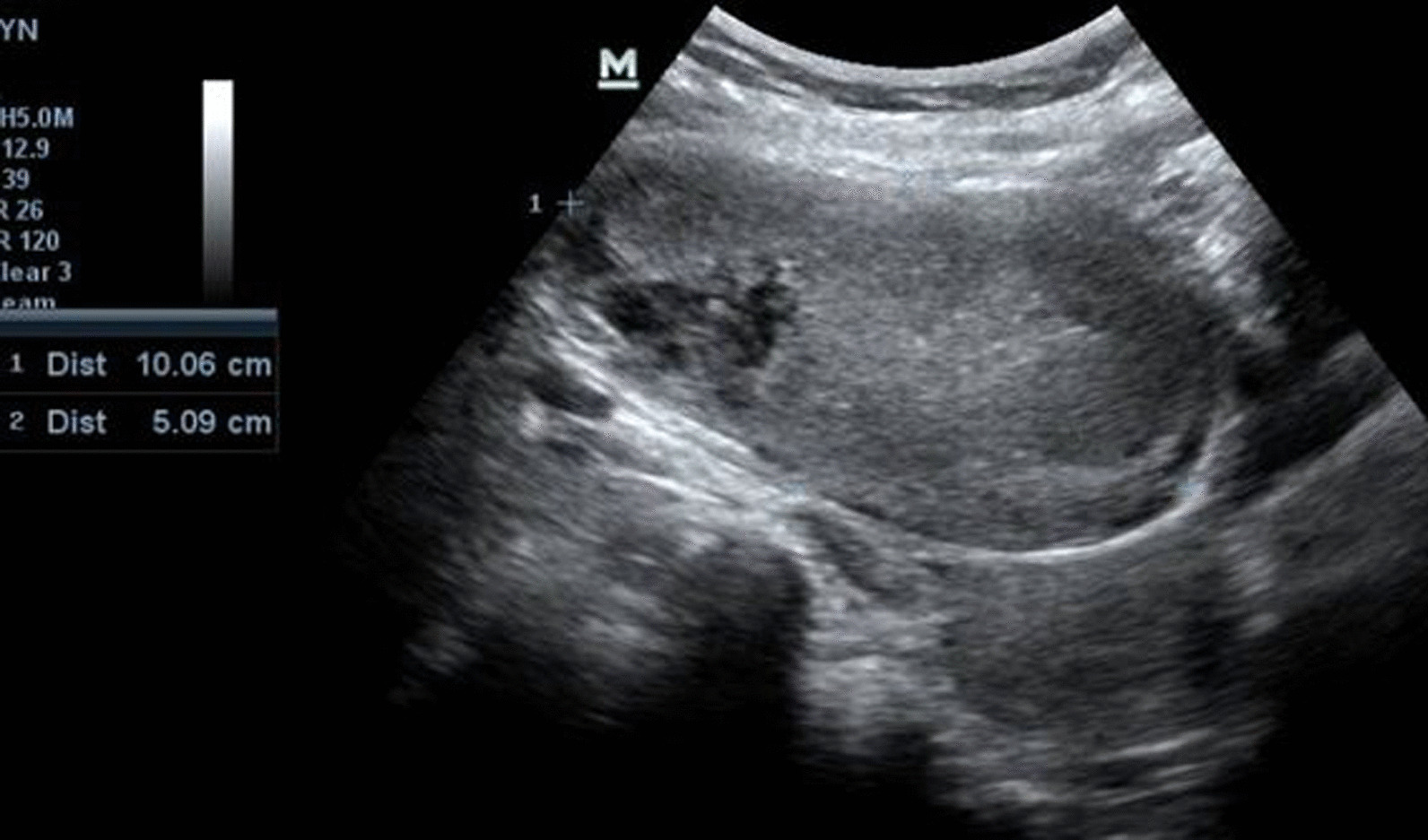
Ultrasonography image showing a well-defined cystic and solid lesion with areas of calcification

The hematological and biochemical parameters, including blood glucose and thyroid levels, were within normal limits. The urine examination was also normal. Ultrasonography revealed a 10 × 5 cm sized, well-defined solid cystic lesion with calcification in the right iliac fossa suggestive of right ovarian dermoid cyst. On Doppler, there was no abnormal flow (Fig. 1).

**Fig. 2:**
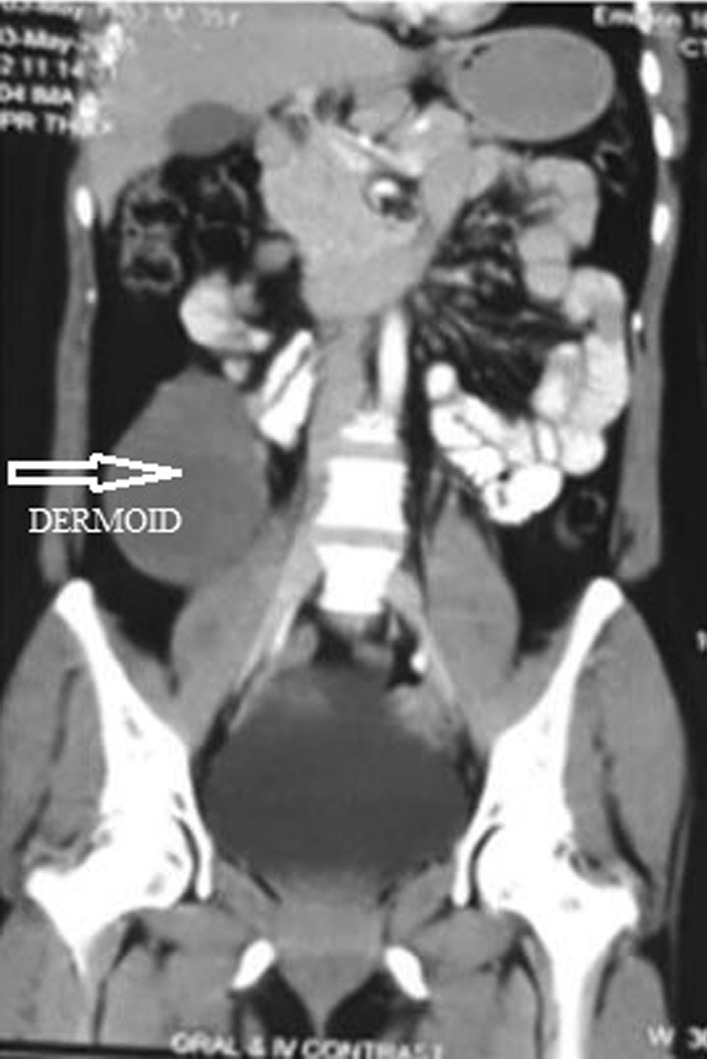
Well-defined hypodense focal lesion in the right lower abdomen and upper pelvis

CECT showed a well-defined hypodense lesion of size 10 × 7 × 5 cm in the right lower abdomen (Fig. 2). The tumor markers CA-125 (8.8 U/ml), carcinoembryonic antigen (1.58 ng/ml), alfa fetoprotein (3.25 ng/ml), lactate dehydrogenase (283 U/l), and inhibin B (29.11 pg/ml) were all normal.

**Fig. 3:**
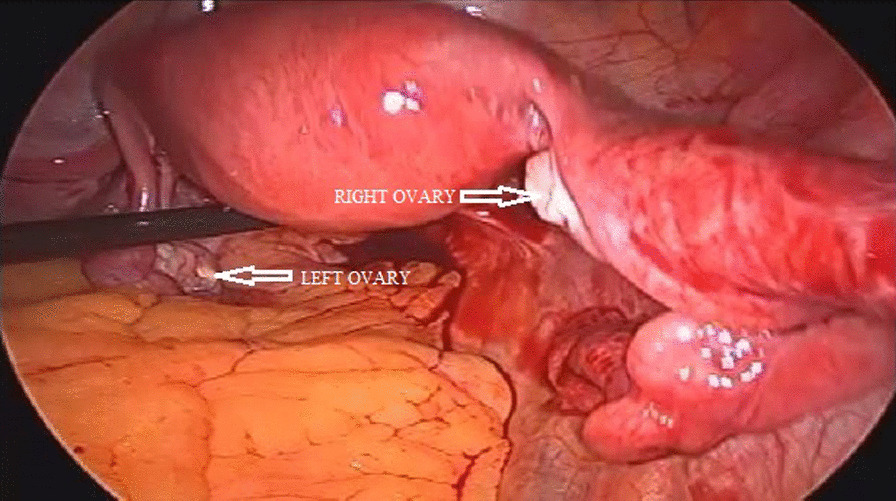
Intraoperative image showing bilateral healthy ovaries

**Fig. 4:**
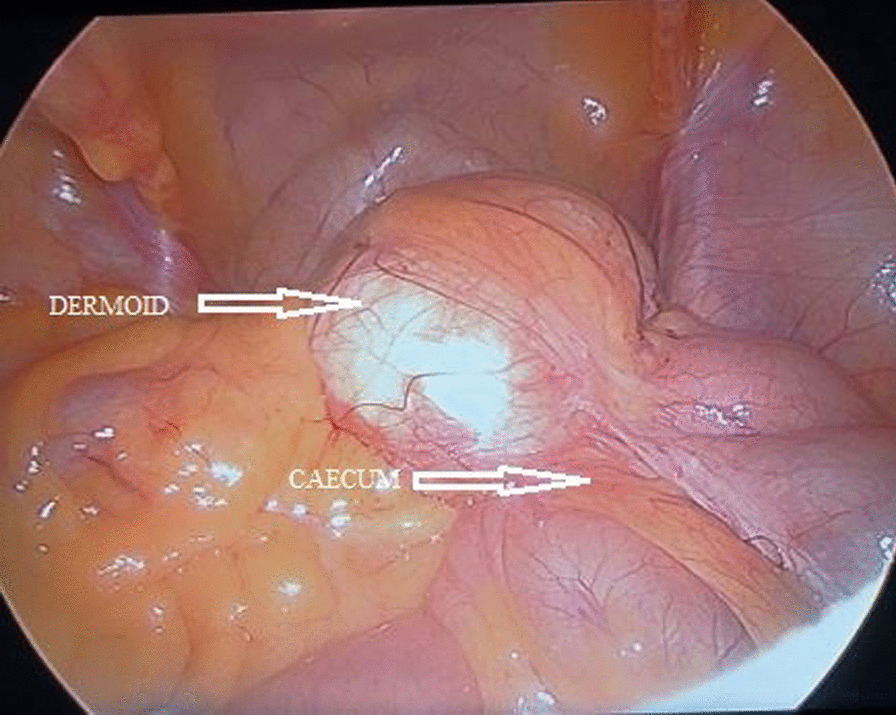
Intraoperative images showing the cyst arising from the cecum

On laparoscopy, the uterus, bilateral tubes, and ovaries appeared normal. A cyst of size 10 × 6 cm was seen arising from the right medial wall of the cecum, at the ileocecal junction. The help of a surgeon was sought intraoperatively, and laparoscopic excision of the cecal dermoid was performed (Figs. 3, 4). The patient had an uneventful postoperative recovery.

**Fig. 5:**
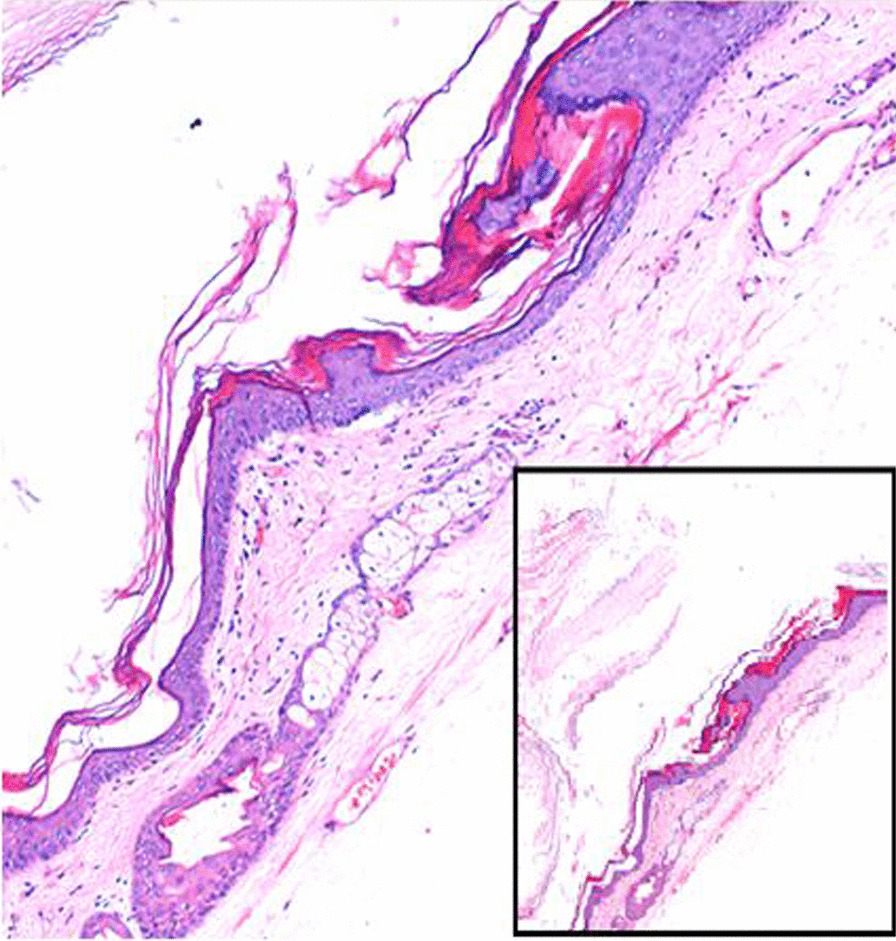
Histopathology showing the squamous epithelium with the presence of a pilosebaceous unit in the subepithelium (hematoxylin and eosin, ×100). The inset showing keratin flakes with stratified squamous epithelium (×4 magnification)

Histopathological study revealed the cyst to be a mature cystic teratoma (Fig. 5). The patient is doing well and has attended regular follow-up for nearly two years now.

## Discussion and conclusion

The word “teratoma,” initially coined by Virchow in 1863, originates from the Greek word* teraton*, meaning monster. The term “dermoid cyst” used much earlier for the same entity was coined by Leblanc, and both descriptors are often used interchangeably [1].

Dermoid cysts can be classified as congenital or acquired. Acquired dermoid cysts may result from previous intra-abdominal surgery or trauma as a result of the seeding of the cutaneous tissue into the peritoneal cavity. Congenital dermoid cysts, however, have been postulated to occur as a result of implantation of the ectoderm during embryogenesis, when the neural groove closes [3].

Mature cystic teratomas commonly originate from the totipotent cells of the mature gonad. Rarely, germ cells which migrate in embryonic life can implant anywhere along the midline or paraxial regions of the body to give rise to extragonadal teratoma. While the omentum is the most common site, the cecum is a rare site for extragonadal teratoma [4].

The exact causes of extragonadal teratoma are yet to be ascertained. Thornton proposed the theory of autoamputation of teratomas from an ovarian site reimplanting to another site in the abdomin [5]. This theory is widely accepted for the origin of extragonadal teratomas in the abdominal cavity. Rarely, the tumor may become entirely detached from the pedicle and become a parasitic teratoma. The mechanisms of autoamputation are still unclear. However, adnexal torsion is believed to lead to infarction, necrosis, and autoamputation. According to another theory, the extragonadal teratomas may occur in an ectopic ovary, which may arise congenitally or following surgery or pelvic inflammation. A third theory suggests that they may originate from displaced primordial germ cells, which later may stop differentiating [5]. However, some authors have criticized these theories as the location of the cecum is off the midline when the neural groove or other epithelial fusion occurs. Cecum, however, is one of the last elements to re-enter the abdomen during the process of intrauterine rotation, at which time it could be taking up some of the congenital heterotopic tissue, which can give rise to the dermoid in the cecum [3].

Cecal dermoid cysts are rare entities, unlike the dermoid cysts from the gonads and other midline structures like the mediastinum, anterior neck, and central nervous system [6]. Continued reports of these cases will help understand and support the theories behind their origin, presentation, and classification.

There have been only ten cecal dermoid cases reported to date. Full-text literature could be retrieved for eight, but the remaining reports were in different languages. The details of these cases are listed in Table 1. Our case seems to be the first reported case of laparoscopic cecal dermoid cystectomy to the best of our knowledge. Although adequate radiological workup, including ultrasonography and computed tomography, was done in our case, the correct site of origin of the dermoid cyst remained elusive until the laparoscopy. The diagnostic dilemma was similar in all the previously reported cecal dermoids, where ascertaining the exact organ of origin was a problem. Excision by laparotomy and laparoscopy seems to be an effective form of curative treatment.

**Table 1:** List of all the cecal dermoids reported to date

Case no.	Age (years)	Sex	Author/reference	Symptom	Tumor size	History of previous surgery	Procedure	Year reported
1	1	F	Kay [7]	Abdominal mass	8 cm	No	Terminal ileum and cecum resected	1971
2	53	F	Mossey [8]	Melena	4 cm	Hysterectomy appendectomy	Laparotomy—right hemicolectomy	1977
3	34	F	Wilkinson [9]	Colicky lower abdomen pain	10 cm	3 cesarean sections	Laparotomy—right hemicolectomy	1996
4	39	F	Mellado [10]	Abdominal distension and pain	20 cm	No	Laparotomy—right hemicolectomy	2000
5	34	M	Nirenberg [11]	Pain lower abdomen	7.5 cm	No	Laparotomy—cystectomy with appendectomy	2001
6	30	M	Schuetz [6]	Pain right lower abdomen	8 cm	No	Laparotomy—right hemicolectomy	2002
7	41	F	Nahidi [12]	Heaviness and mass	10 cm	No	Laparotomy—right Mikulicz colostomy	2016
8	2	M	Destro [13]	Abdominal distension	6 cm	Anorectal malformation surgery	Laparotomy—left hemicolectomy	2019
9	35	F	Our case	Pain right lower abdomen	10 cm	No	Laparoscopic cystectomy	2020

yrs = years, M = Male, F = Female, cm = centimetres

This case emphasizes the need for an accurate preoperative diagnosis to avoid intraoperative surprises, as happened to the gynecologists. Cecal dermoid should be kept in mind as a possibility wherever a diagnostic dilemma exists. Laparoscopy is not only feasible in the management of this condition but also carries a promise of lesser morbidity and early recovery.

## Data Availability

Not applicable.

## References

[1] Comerci JT, Licciardi F, Bergh PA, Gregori C, Breen JL. Mature cystic teratoma: a clinicopathologic evaluation of 517 cases and review of literature. Obs Gynecol. 1994;84(1):22–8. 8008317

[2] Khanna S, Srivastava V, Saroj S, Mishra SP, Gupta SK. An unusual presentation of ovarian teratoma: a case report. Case Rep Emerg Med. 2012;2012:1–2. 10.1155/2012/845198 PMC3542945 23326728

[3] Lao VV, Stark R, Lendvay TS, Drugas GT. Cecal dermoid cyst. J Pediatr Surg Case Rep. 2014;2(7):347–9. 10.1016/j.epsc.2014.06.015.

[4] Hegde P. Extragonadal omental teratoma: a case report. J Obstet Gynaecol Res. 2014;40(2):618–21. 10.1111/jog.12198 24147579

[5] Lee KH, Song MJ, Jung IC, Lee YS, Park EK. Autoamputation of an ovarian mature cystic teratoma: a case report and a review of the literature. World J Surg Oncol. 2016;14(1):1–7. 10.1186/s12957-016-0981-7. PMC4989355 27535361

[6] Schuetz MJ, Elsheikh TM. Dermoid cyst (mature cystic teratoma) of the cecum: histologic and cytologic features with review of the literature. Arch Pathol Lab Med. 2002;126(1):97–9. 10.5858/2002-126-0097-DCMCTO 11800659

[7] Kay S. Teratoid cyst of the cecum. Am J Dig Dis. 1971;16(3):265–8. 10.1007/BF02235249 5554506

[8] Mossey JF, Rivers L, Patterson P. Dermoid cyst of the cecum. Can Med Assoc J. 1977;117(12):1372. PMC1880432 589535

[9] Wilkinson N, Cairns A, Benbow EW, Donnai P, Buckley CH. Dermoid cyst of the cecum. Histopathology. 1996;29(29):186–8. 10.1046/j.1365-2559.1996.d01-489.x 8872157

[10] Mellado J, Bosch-Príncep R, Pérez-del-Palomar L. Ultrasonography and CT findings of a dermoid cyst of the cecum: a case report. Acta Radiol. 2000;41(5):489–91. 10.1080/028418500127345767 11016773

[11] Nirenberg A, Buxton NJC, Kubacz GJ. Dermoid cyst of the cecum: case report. Pathology. 2001;33(2):246–7. 11358064

[12] Nahidi A, Jazayeri S, Fathizadeh P. Dermoid cyst of the cecum: case report. Jundishapur J Oncol. 2016;2(1):15–9.

[13] Destro F, Maestri L, Meroni M, *et al.* Colonic mature cystic teratoma. J Pediatr Surg Case Rep. 2019;41:57–9.

